# Ganglioside GM3 Has an Essential Role in the Pathogenesis and Progression of Rheumatoid Arthritis

**DOI:** 10.1371/journal.pone.0040136

**Published:** 2012-06-29

**Authors:** Yukinori Tsukuda, Norimasa Iwasaki, Naoki Seito, Masashi Kanayama, Naoki Fujitani, Yasuro Shinohara, Yasuhiko Kasahara, Tomohiro Onodera, Koji Suzuki, Tsuyoshi Asano, Akio Minami, Tadashi Yamashita

**Affiliations:** 1 Department of Orthopaedic Surgery, Hokkaido University Graduate School of Medicine, Sapporo, Japan; 2 Division of Molecular Immunology, Institute for Genetic Medicine, Hokkaido University, Sapporo, Japan; 3 Laboratory of Medical and Functional Glycomics, Graduate School of Advanced Life Science, Hokkaido University, Sapporo, Japan; 4 Hokkaido Orthopaedic Memorial Hospital, Sapporo, Japan; 5 Faculty of Advanced Life Science, Frontier Research Center for Post-Genomic Science and Technology, Hokkaido University, Sapporo, Japan; 6 World Class University Program, Kyungpook National University School of Medicine, Daegu, Korea; University of Pittsburgh, United States of America

## Abstract

Rheumatoid arthritis (RA), a chronic systemic inflammatory disorder that principally attacks synovial joints, afflicts over 2 million people in the United States. Interleukin (IL)-17 is considered to be a master cytokine in chronic, destructive arthritis. Levels of the ganglioside GM3, one of the most primitive glycosphingolipids containing a sialic acid in the structure, are remarkably decreased in the synovium of patients with RA. Based on the increased cytokine secretions observed in *in vitro* experiments, GM3 might have an immunologic role. Here, to clarify the association between RA and GM3, we established a collagen-induced arthritis mouse model using the null mutation of the ganglioside GM3 synthase gene. GM3 deficiency exacerbated inflammatory arthritis in the mouse model of RA. In addition, disrupting GM3 induced T cell activation *in vivo* and promoted overproduction of the cytokines involved in RA. In contrast, the amount of the GM3 synthase gene transcript in the synovium was higher in patients with RA than in those with osteoarthritis. These findings indicate a crucial role for GM3 in the pathogenesis and progression of RA. Control of glycosphingolipids such as GM3 might therefore provide a novel therapeutic strategy for RA.

## Introduction

Rheumatoid arthritis (RA) is an autoimmune disease characterized by chronic inflammation of the synovial tissues in multiple joints, leading to joint destruction [1]. The pathologic features of RA include hyperplasia of the synovial lining cell layer; infiltration of inflammatory cells in the subintima, comprising predominately lymphocytes, plasma cells, and macrophages; and deposition of fibrin on the synovial surfaces, especially in clinically active disease. The pathogenesis of RA, however, is not fully understood. CD4^+^ T cells, key molecules in primary inflammatory lesions, have an essential role in the initiation of subsequent inflammatory responses [2]. In particular, Th17 cells (a subset of CD4^+^ T cells that are distinct from Th1 and Th2) and regulatory T cells are suggested to mediate inflammation and thus have a key role in the pathogenesis of RA [3]. Furthermore, interleukin (IL)-17, secreted by Th17 cells, stimulates the production of IL-6, IL-1, tumor necrosis factor (TNF)α, IL-8, matrix metalloproteinases, and other proinflammatory factors [4]. The cytokine IL-17 enhances the inflammation associated with RA and contributes to the pathogenesis of RA by inducing monocyte migration into the inflamed synovial tissue [5], [6]. High-level production of proinflammatory cytokines, such as IL-1 and TNFα, in the synovium results from an interaction between monocytes or macrophage cells and synoviocytes [7]. The regulatory mechanism of Th17 cells in RA, however, remains unclear.

**Figure 1:**
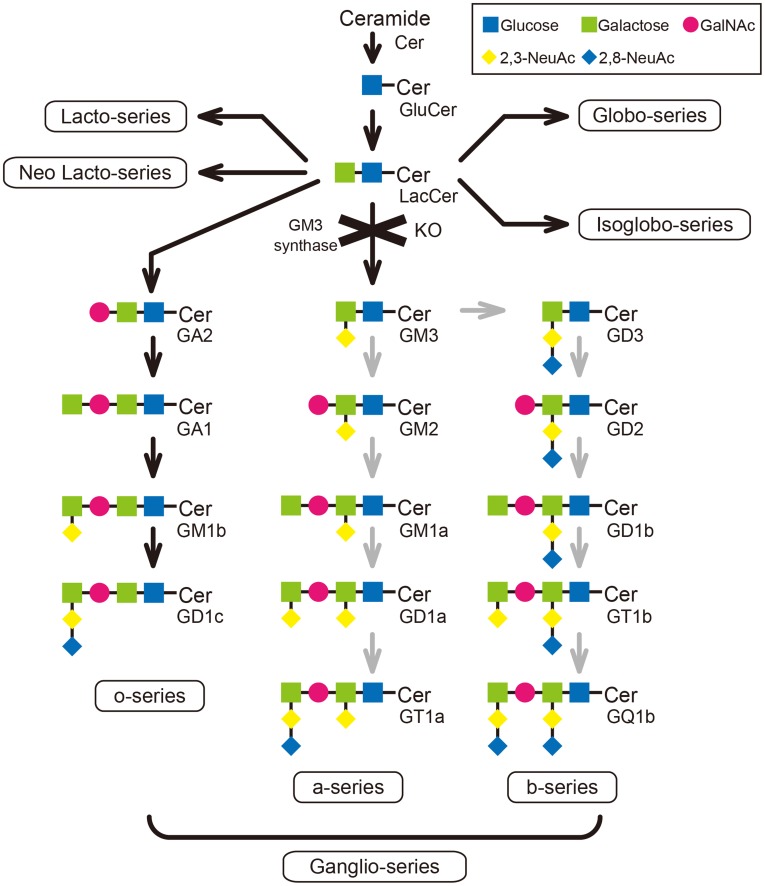
Lactosylceramide (LacCer) is the key branching point of sphingolipid biosynthesis. In the synthetic pathway of sphingolipids, four alternate pathways (lacto-series, neolacto-series, globo-series, and isoglobo-series) branch-off from LacCer. GM3S^−/−^ mice, in which the GM3 synthase gene is disrupted, produced only o-series gangliosides, including GA2 and GA1, and not a-series or b-series gangliosides.

Ganglioside GM3 and its derivatives (Figure 1) are membrane-bound glycosphingolipids (GSLs) composed of an oligosaccharide head structure containing one or more sialic acid residue [8]. GSLs act to transduce signals involved in cell surface events, including the phosphorylation of transmembrane receptors [9]. GM3 is the most widely distributed ganglioside among tissues, and serves as a precursor for most of the more complex ganglioside species [10]. GM3 inhibits the function of fibroblast growth factor receptor [11], and cell growth is regulated by GM3-enriched microdomain [12]. GM3 is thought to inhibit immunologic functions, such as the proliferation and production of cytokines by T cells [13]. In contrast, higher levels of GM3 in lipid rafts promote an increase in the T cell responsiveness to stimulation *in vitro* [14]. Few studies, however, have assessed whether the immunoreaction related to gangliosides occurs as a positive or negative event *in vivo*. T cells are the predominant infiltrating lymphocytes in the synovium of RA patients [15]. Thus, we hypothesized that GM3 is involved in the T cell mechanism of RA, thereby contributing to the clinical features of RA. The aims of the present study were to determine the relation betweenGM3 and the pathogenesis or progression of RA, and to clarify the effect of GM3 on Th17 cell proliferation and IL-17 secretion from Th17 cells using a mouse collagen-induced arthritis (CIA) model.

## Results

### GM3 is Increased in the Synovium of RA Patients

**Figure 2:**
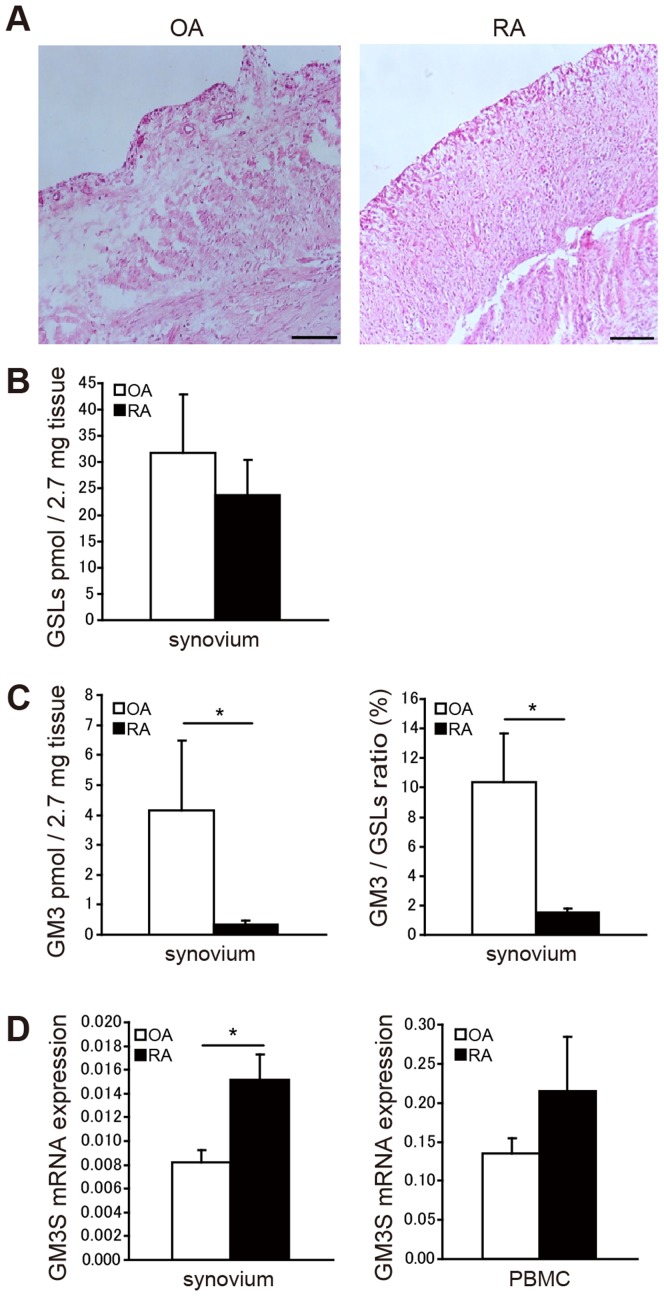
Clinical features related to GM3 in patients with RA or OA. A. Histology of RA and OA patients. Hematoxylin and eosin (HE) staining. Scale bars  = 200 µm. **B.** Quantification of total GSL glycans in human synovium (*n*  = 5 per group). **C.** Absolute and relative amounts of GM3 glycans in human synovium (*n*  = 5 per group). **D.** Quantification of GM3S mRNA in synovium and PBMCs in RA and OA patients (*n*  = 5 per group). Data shown are mean ± SEM. *P<0.05, compared to OA patients.

Infiltration of multi-stratified synoviocytes and inflammatory cells into synovial tissues is commonly observed in RA patients (Figure 2A). We used mass spectrometry to investigate the amount of GSL in the synovium. The total GSL amount was slightly decreased in RA patients (Figure 2B). The amount of GM3 in the ganglioside synthetic pathway was decreased in the synovium of RA patients compared to osteoarthritis (OA) patients (Figure 2C). The relative amount of GM3 among GSLs was also lower in RA patients than in OA patients (Figure 2C). In contrast to the amount of GM3 production in the synovium, GM3 synthase (GM3S) mRNA transcription was increased in the synovium and in the peripheral blood mononuclear cells (PBMCs) of RA patients (Figure 2D). These results suggest that GM3 may be involved in the pathogenesis of RA.

### Higher GM3 Synthase mRNA Gene Expression in CIA

**Figure 3:**
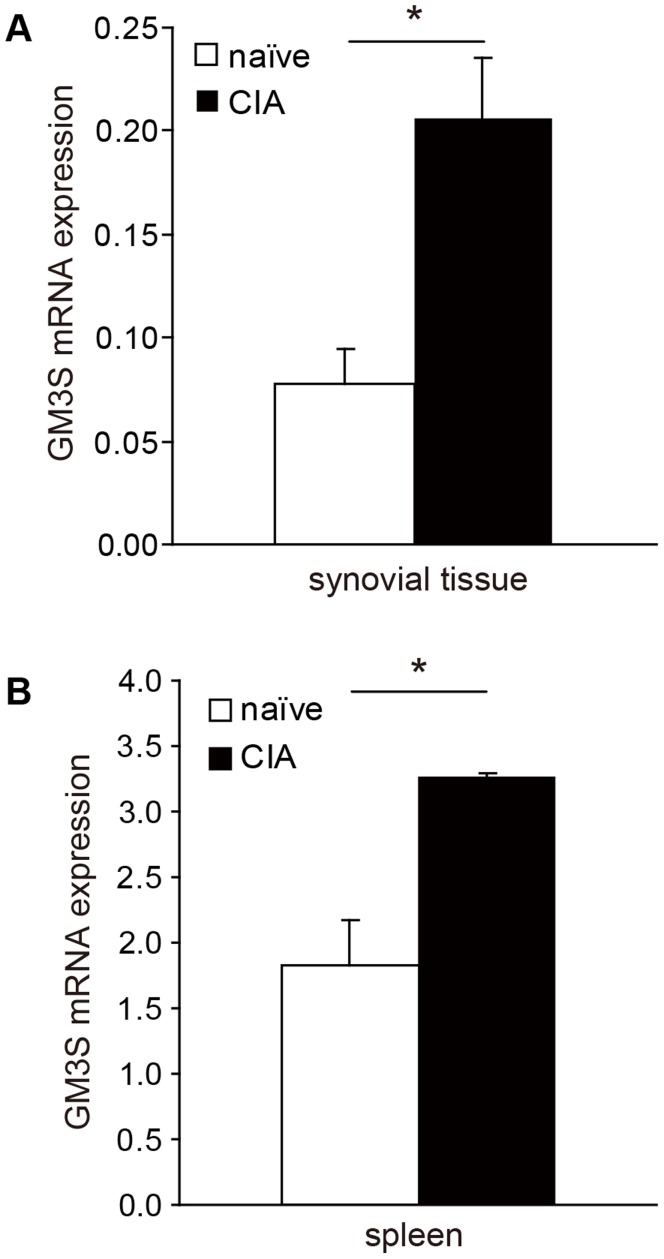
Quantification of GM3S mRNA in synovial tissues and spleens from C57BL/6 naïve and CIA mice. Mice were anesthetized and killed on day 35 after CII-CFA stimulation and synovial tissues and spleens were removed to analyze GM3S mRNA (*n*  = 5 per group). **A.** mRNA in synovial tissue. **B.** mRNA in spleen. Data shown are mean ± SEM. *P<0.05, compared with naïve WT mice.

To determine whether GM3 is involved in the pathogenesis of RA, we selected a CIA model. The CIA model is a well-established model suitable for extrapolation to human RA [16]. At 35 days after immunization with chick collagen type II (CII), GM3S mRNA transcription was increased in the synovial tissue and spleen of CIA wild-type (WT) mice compared with naïve WT mice (Figure 3). This finding suggests that inflammation derived from CIA leads to an increase in GM3 synthase gene expression.

### The Lack of GM3 Accelerates the Pathogenesis and Severity of CIA

**Figure 4:**
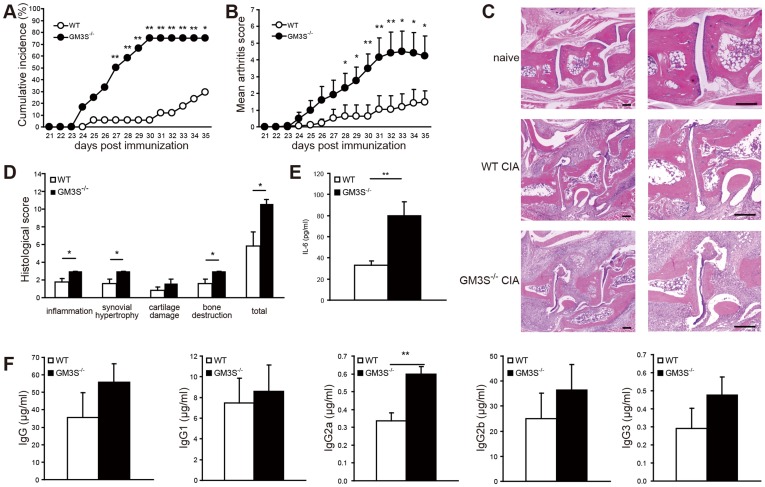
Phenotype of WT and GM3S^−/−^ CIA mice. CIA was induced in C57BL/6 WT and GM3S^−/−^ mice (10–15 weeks old). Mice were immunized with chicken CII emulsified with CFA on days 0 and 21(*n*  = 17 WT and *n*  = 12 GM3S^−/−^ mice). **A.** Cumulative incidence of arthritis. **B.** Arthritis score with CII-CFA. **C.** Photomicrographs show HE-stained paraffin sections of the right hind limbs of naïve, CIA WT, and CIA GM3S^−/−^ mice at day 35 of the study. Scale bars  = 200 µm **D.** Histological scores on day 35 after primary immunization with CII-CFA (*n*  = 5 per group). **E.** Serum IL-6 levels in mice on day 25 after primary immunization with CII-CFA (*n*  = 5 per group). **F.** Serum levels of total IgG, IgG1, IgG2a, IgG2b, and IgG3 anti-chicken CII antibodies with CII-CFA on day 35 after primary immunization (*n*  = 5 per group). Data shown are mean ± SEM. *P<0.05, **P<0.01 compared to WT mice.

To investigate the relationship between GM3 and the development of autoimmune arthritis, we used knockout mice with disruption of the GM3 synthase gene (EC 2.4.99.9; GM3S^−/−^ mice) [8]. The incidence of arthritis and arthritis scores of the GM3S^−/−^ mice were significantly greater than those of WT mice (Figure 4A and B). Although the genetic background of the mice influences the arthritis score (i.e., C57BL/6 is a low responder strain for the CIA model [17]), GM3S^−/−^ mice with a C57BL/6 genetic background had a greater response. Two weeks after secondary immunization, morphologically progressive destruction, synovial invasion, and a large number of inflammatory cells were observed in the joint region in GM3S^−/−^ mice. Based on the histological scoring, GM3S^−/−^ mice exhibited significantly worse inflammation, synovial hypertrophy, and bone destruction compared with WT mice (Figure 4C and D). At 25 days after immunization, serum levels of IL-6 as an exacerbation factor of RA, were significantly higher in GM3S^−/−^ mice than in WT mice (Figure 4E). CII-specific serum IgG2a levels at 35 days after immunization were significantly higher in GM3S^−/−^ mice than in WT mice (Figure 4F). The onset was earlier in GM3S^−/−^ mice than in WT mice (Table 1). These results confirm that GM3 deficiency leads to the development of CIA, and accelerates the pathogenesis of CIA.

**Table 1:** Onset of CIA from primary immunization.

	WT	GM3S^−/−^
onset	31.6±1.8	26.7±0.7*

Data shown are mean ± SEM. *P<0.05, compared to WT mice.

### GM3 Deficiency Promotes the Proliferation of Th17 Cells in CIA Models

**Figure 5:**
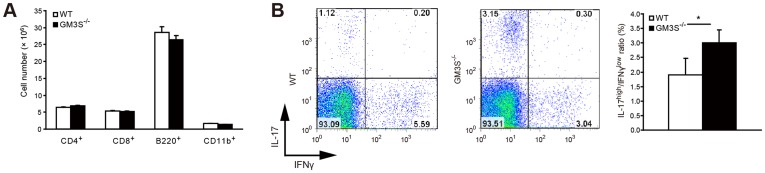
Immune response of T cells in WT and GM3S^−/−^ mouse CIA models. A. CD4^+^, CD8^+^, B220^+^, and CD11b^+^ T cell contents of iLNs in WT and GM3S^−/−^ mice on day 7 after immunization with CII-CFA (*n*  = 3 per group). **B.** The Th17 cell content in CD4^+^ T cells in mouse iLNs (*n*  = 3 per group). Data shown are mean ± SEM. *P<0.05, compared to WT mice.

These suppressive roles of GM3 in the development of CIA suggest that GM3 may be involved in CD4^+^ T cell behaviors because CD4^+^ T cells are the predominant infiltrating lymphocytes in RA synovium [15]. Therefore, we investigated the T cell proliferation in GM3S^−/−^ CIA mice. Based on flow cytometry analysis, the numbers of CD4^+^/CD8^+^ T, B220^+^, and CD11b^+^ cells in the inguinal lymph nodes (iLNs) 1 week after CII stimulation were not significantly different (Figure 5A). The ratio of Th17 cells in CD4^+^ T cells, on the other hand, was significantly increased in GM3S^−/−^ mice (Figure 5B). These findings suggest that the loss of GM3 induces the proliferation of Th17 cells as a CD4^+^ T cell subset under CII stimulation.

### Deletion of GM3 Promotes High Immunologic Response of T Cells

**Figure 6:**
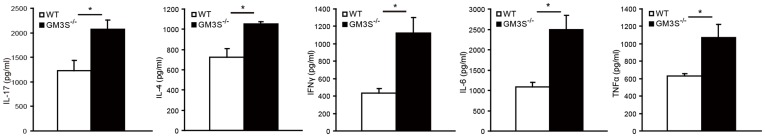
T-cell immune response following anti-CD3 antibody stimulation. Serum levels of IL-17, IL-4, IFNγ, IL-6, and TNFα at 1.5 h after intraperitoneal administration of 20 µg anti-CD3 antibody (*n*  = 5 per group). Data shown are mean ± SEM. *P<0.05, compared to WT mice.

To investigate the immunologic response of T cells by the deletion of GM3 *in vivo*, anti-CD3 antibody was intraperitoneally administered to mice and IL-17, IL-4, interferon (IFN) γ, IL-6, and TNFα levels in serum were measured. These cytokine levels were higher in GM3S^−/−^ mice than in WT mice (Figure 6). These findings indicate that GM3 may act as a regulatory molecule to suppress the immunologic response in CD4^+^ T cells to stimulus of T cell receptor antigen, thereby the lack of GM3 may lead to high levels of cytokines, especially IL-17 secretion from Th17 cells.

## Discussion

Gangliosides containing one or more sialic acid residues are present in all mammalian cell plasma membranes and intracellular membrane structures [18]. Gangliosides have essential roles in the early development, cell differentiation, proliferation, and the immune response [19]. GM3 is the most widely distributed ganglioside among tissues, and serves as a precursor for most of the more complex ganglioside species [10]. The role of GM3 in RA, however, is not almost clear. In the present study, the total GSL amount was slightly decreased in RA patients. The amount of GM3, on the other hand, was significantly decreased in the synovium of RA patients compared to OA patients. GSLs comprise many derivatives starting from ceramide, and five distinguishable synthetic pathways from lactosylceramide have been confirmed (Figure 1). Therefore, it is reasonable that the total GSL amount was not remarkably different, despite the fact that GM3 alone was decreased in the synthetic pathways. In contrast, GM3S mRNA transcription was increased in the synovium and in the PBMCs of RA patients. Therefore, the amount of GM3 detected by mass spectrometry revealed a gap between GM3S mRNA transcription and GM3 production. RA patients likely have a mechanism to compensate for the lack of GM3.

We investigated whether GM3 has a suppressive role in the development of autoimmune arthritis in a CIA model using GM3S^−/−^ mice. The development of CIA was significantly higher in GM3S^−/−^ mice. Th17 cells produce various cytokines including IL-17. IL-17 has profound pro-inflammatory effects and induces tissue damage during the course of various autoimmune diseases, such as the CIA animal model [20], [21]. Th17 cells are distinct from Th1, Th2, and regulatory T cells. IL-17 causes cartilage and bone destruction in mice [22]. Mice deficient in IL-17 are resistant to the pathogenesis and progression of CIA [1]. In humans, IL-17 directly causes cartilage and bone destruction in RA patients [23]. IL-17 stimulates the production of IL-6, TNFα, and other proinflammatory factors [4]. The differentiation of Th17 cells is dependent on IL-6 [24]. IL-17 activates synovial fibroblasts and macrophages to stimulate the production of IL-6 and TNFα, which lead to joint inflammation [25], [26]. In the present study, the ratio of Th17 cells in CIA was significantly higher in the iLN of GM3S^−/−^ mice. Furthermore, serum IL-6 levels were significantly higher in GM3S^−/−^ mice. Our results are consistent with these previous reports and suggest that the augmentation of Th17 cells in GM3S^−/−^ mice induces a greater increase in the serum IL-6 levels than in WT mice. Our findings that GM3S^−/−^ mice secrete more IL-17 under CIA stimulation are also consistent with the fact that IL-17 is involved in the initiation and progression of CIA, and that inhibition of IL-17 in a mouse CIA model reduces inflammation and bone erosion [27], [28]. Based on these findings, GM3 suppresses the Th17 cell proliferation in autoimmune arthritis. These findings confirm some previous reports [13], [29]. In contrast, however, higher levels of GM3 in lipid rafts are reported to promote an increase in naïve T cell responsiveness to stimulation in *in vitro* culture [14]. In the *in vivo* Th-17 disease model, GM3S*^−/−^* mice had more severe arthritis and expressed larger quantities of cytokines. Thus, it remains unclear whether GM3 affects the disease pathogenesis. We could not find previous studies according to the relation between GM3 and RA, and diseases which GM3 decreases. At the present time, the mechanism accelerating the development of CIA by the deletion of GM3 is unknown. However, based on our results, two factors are the suspected causes. First factor is the high Th17 cell augmentation. Second factor is higher susceptibility to stimulation of T cells, especially Th17 cells, due to GM3 deficiency in a CIA model. To confirm this susceptibility, we investigated whether GM3 is involved in the reaction to the T cell receptor antigen, anti-CD3 antibody. The levels of IL-17, IL-4, IFNγ, IL-6, and TNFα in the serum were higher in GM3S^−/−^ mice than in WT mice. These findings suggest that GM3 is involved in the T cell susceptibility to stimulation. By these reasons, it is possible that IL-17 production and secretion are increase in a GM3 deficiency CIA model.

To our knowledge, this is the first study to show a relation between GM3 and the pathogenesis and progression of RA and CIA *in vivo*. The present findings indicate that GM3 may become a regulatory molecule against the production and secretion of cytokines, especially IL-17 derived from Th17 cells, which contributes to the pathogenesis and progression of RA. RA synovial fibroblasts are targeted by IL-17 in the joint synovium [30]. IL-17 enhances the release of IL-6, which is a key inflammatory cytokine in the joints [31], [32]. We produced T cell-specific knockout mice with a null mutation of the glucosylceramide synthase (*Ugcg*). The T cells in these mice do not contain any GSL. We assessed serum IL-17 levels after administrating anti-CD3 antibody and CIA severity. Serum IL-17 levels in T cell-specific mice after administrating anti-CD3 antibody were almost the same as those in GM3S^−/−^ mice. Interestingly, the CIA incidence and arthritis scores in GM3S^−/−^ mice were very severe, but the severity in T cell-specific mice was milder than that in GM3S^−/−^ mice (data not shown). Consequently, our results suggest that GM3 may be involved not only in cytokine production but also in the sensitivity of joint synovium.

In this study, there are some limitations. First, the mechanism of developing CIA in GM3 deficiency is unknown. GSLs have been implicated as inhibiting various signal transductions on cell membranes [33]. As one of the possibility, GM3 may be involved in inhibiting the signal transduction on Th17 cells in CIA. Second, The relation between GM3 and cells except T cells (macrophage, osteoclast, fibroblast, or synoviocyte and so on) in CIA is not clear. To our knowledge, there are no reports of this relation. Third, GM3S^−/−^ mice have O-series gangliosides different from a- or b-series gangliosides, and O-series gangliosides may compensate for the complete absence of the products of GM3S in GM3S^−/−^ mice [34]. In the results from our MS analysis, O-series gangliosides did not show the significant increase of their amounts in synovium. It is a strong possibility that another pathways (Lacto, Neolacto, Globo, and Neoglobo) through the lactosylceramides may increase their products. Due to the samples of gangliosides derived from fibroblast and synovium, any products with abnormal structures which are not normally expressed in wild type were not detected in synovium. However, to do the complete detection of their products, we should examine MS/MS analysis and more. We will investigate the relation between o-series gangliosides and CIA, because O-series gangliosides have the potential to make an effect on the pathogenesis or progression of CIA in GM3S^−/−^ mice. Finally, we speculate that GM3 strongly participate in the pathogenesis and progression of human RA. Further studies are required to confirm our speculation.

In conclusion, our findings indicate that GM3 deletion accelerates the pathogenesis and progression of RA and mouse CIA by increasing IL-17 proliferation and secretion from Th17 cells. Based on these findings, we reasonably conclude that GM3 may act as a regulatory molecule to suppress the proliferation and secretion of IL-17 from Th17 cells, thereby preventing the pathogenesis and progression of RA and CIA. GM3 might therefore be a factor for novel and effective strategies for the treatment of RA.

## Materials and Methods

### Ethics Statement

Written informed consent was obtained from all the participants after being approved by the Ethics Committee of the School of Medicine, Hokkaido University (permit number: 008–0076). All experimental protocols of mice were in compliance with the rules and regulations of the Animal Care and Use Committee of Hokkaido University (permit number: 23 (26) ). All experiments were performed in Hokkaido University.

### Patients

Synovium was obtained from five patients with RA and five patients with OA undergoing total knee arthroplasty, and blood was obtained from all patients during the postoperative period. The RA patients fulfilled the criteria of the American College of Rheumatology.

### Mice

GM3 synthase knockout (GM3S^−/−^) mice were generated as described previously [8]. Male GM3S^−/−^ mice (10–15 weeks old) were backcrossed over 15 generations to C57BL/6 mice, and age-matched male C57BL/6 wild-type (WT) mice were used as a control.

### Cell Preparation

PBMCs were isolated from the heparinized blood of patients with RA or OA. The cells were collected using Ficoll-Paque (GE Healthcare) density-gradient centrifugation. Spleens and iLNs in mice were isolated and passed through cell strainers (BD Falcon) to obtain single-cell suspensions. Red blood cells isolated from the spleen were lysed by two consecutive incubations (5 and 3 minutes at 37°C) in an NH4Cl suspension (0.83% in 0.01 M Tris-HCl, pH 7.2). The remaining cells were washed. These cells were used for real-time reverse transcription-polymerase chain reaction (PCR) and flow cytometry.

### RNA Isolation and Real-time Reverse Transcription-PCR

We isolated total RNA in the synovium and PBMCs from humans, and synovial tissues and splenocytes in mice using Trizol reagent and analyzed the RNA for GM3 synthase gene expression. RNA expression was determined by absorbance at wavelengths of 260 nm and 280 nm. Total RNA (1 µg) was converted to cDNA using ImPromIITM Reverse Transcriptase (Promega) according to the manufacturer’s instructions. We performed quantitative analysis of the mRNA levels using Opticon Real-time PCR (Bio-Rad Laboratories) with sequence-specific primer pairs: human GM3S; (5′-3′) GAACTCTTGCCAGAGCACGA (3′-5′) GACGTGCCTAATCTTGACCC, mouse GM3S; (5′-3′) ATGCCAAGTGAGTTCACCTCT (3′-5′) GTGCAACCAACGTAAACCTCA, human GAPDH; (5′-3′) TTCCATGGCACCGTCAA (3′-5′) ACTCATGCAGCACCTCAGGT, mouse GAPDH; (5′-3′) ACTTTGTCAAGCTCATTTCC (3′-5′) GTAGTTATTTCAAGCGACGT. The PCR was performed in triplicate on cDNA samples using SYBR Green Master Mix (Finzymes) according to the manufacturer's protocol. GM3 synthase gene expression levels were normalized to those of glyceraldehyde 3-phosphate dehydrogenase in each sample.

### Mass Spectrometry

GSLs were extracted from human synovium. GSL glycans were digested by enzymatic reaction employing GSL-specific endoglycoceramidases II (EGCase II) derived from mutant strain M-750 of *Rhodococcus* sp. (TAKARA Bio). The solutions treated with EGCase II were subjected to glycoblotting using a protocol similar to that used for *N*-glycome analyses reported previously [35]. The samples were analyzed by Matrix Assisted Laser Desorption Ionization - Time of Flight (TOF) MS on an Ultraflex II TOF/TOF mass spectrometer (Bruker Daltonics) equipped with a reflector, and controlled by the FlexControl 3.0 software package (Bruker Daltonics). Peaks were detected as proton-adducted ions. Masses were annotated using the FlexAnalysis 3.0 software package (Bruker Daltonics). In addition to the TOF/TOF analyses, we used the GlycoSuiteDB (http://glycosuitedb.expasy.org/glycosuite/glycodb) and SphinGOMAP^©^ (http://www.sphingomap.org/) databases to identify the glycan structures.

### Collagen-induced Arthritis

CIA was induced in C57BL/6-background WT or GM3S^−/−^ mice as described previously [36]. Briefly, CIA was induced using an emulsion comprising equal amounts of Complete Freund’s Adjuvant (CFA) containing 5 mg/ml heat-killed *Mycobacterium tuberculosis* (Chondrex) and chick type II collagen (CII) solubilized at 2 mg/ml in 0.05 M acetic acid (Chondrex). The mice were immunized with 100 µl of emulsion (100 µg chick CII) intradermally at several sites at the base of the tail. After 21 days of primary immunization, the mice received intradermal boosters with a volume of emulsion equal to that of the primary immunization near the primary injection sites.

### Clinical Assessment of Arthritis

The mice were examined daily for clinical symptoms of arthritis, and a mean arthritis score was calculated as described previously [37]: score 0, normal; score 1, redness and/or swelling in one joint; score 2, redness and/or swelling in more than one joint; score 3, redness and/or swelling of the entire paw; score 4, deformity and/or ankylosis.

### Histological Assessment of Arthritis

For histological examination of joints, mice were anesthetized and killed at day 35 after primary immunization. The right hind paw was dissected and placed in 10% buffered formalin and decalcified in 10% EDTA, pH 7.5, for 1 week. These tissues were then dehydrated in an ascending series of ethanol, the tissues were embedded in paraffin, sectioned, and stained with hematoxylin and eosin (HE). Sections were assessed using a separate histological score of 0 (normal), 1 (mild), 2 (moderate), or 3 (severe) for inflammation, synovial hypertrophy, cartilage damage, and bone destruction [38]. Human synovial tissues were obtained from patients with total knee arthroplasty and immediately embedded in Optimal Cutting Temperature (OCT) compound for frozen sections. Sections (5 µm thick) were cut from frozen tissue samples, fixed in acetone, air-dried and used for HE staining.

### Measurement of Total and Anti-CII IgG Antibodies

Blood samples were obtained from the orbital sinus and allowed to clot at room temperature for 1 h. Individual sera were measured for total and anti-CII antibodies (IgG1, IgG2a, IgG2b, and IgG3) on day 35 after primary immunization using an enzyme-linked immunosorbent assay (ELISA) kit (Chondrex) according to the manufacturer’s instructions.

### Flow Cytometric Analysis

We collected iLN from C57BL/6 mice on day 7 after immunization with CII-CFA. Cells were washed and blocked with 1 µg/ml of Fcγ blocker (BD Pharmingen). After washing, to analyze the iLN population of immune cells, the cells were incubated with the indicated fluorescein isothiocyanate (FITC)-, phycoerythrin (PE)-, and allophycocyanin-conjugated antibodies (0.5 µg for 30 min) to stain for CD3, CD4, CD8, CD11b, and B220.

For investigating the ratio of Th17 cells in CD4^+^ T cells, iLN cells were incubated for 5.5 h with phorbol myristate acetate (20 ng/ml), ionomycin (250 ng/ml), and brefeldin A (1 µl/ml; GolgiPlug; BD Biosciences). Cells were washed and blocked with 1 µg/ml Fcγ blocker (BD Pharmingen) before extracellular staining for CD3 and CD4. Cells were then fixed and permeabilized with BD Cytofix/Cytoperm (BD Bioscience) and stained intracellularly for IL-17 or IFNγ. Flow cytometric analysis was performed with FACS Caliber (BD, Mountain View) and Flowjo software (TREESTER). We used the following antibodies: FITC-conjugated anti-CD3 antibody (BioLegend), PE-conjugated anti-CD4 antibody (BioLegend), APC-conjugated anti-CD8a antibody (Pharmingen) PE-conjugated anti-B220 antibody (BioLegend), FITC-conjugated anti-CD11b antibody (BD Pharmingen), PE-conjugated anti-IFNγ antibody (BD Pharmingen), and allophycocyanin-conjugated anti-IL-17 antibody (BioLegend).

### Response of T Cells with Anti-CD3 Antibody in vivo

Individual mice were intraperitoneally administered 20 µg anti-CD3 antibody. Serum was collected 1.5 h later and IL-17, IL-4, IFNγ, TNFα, IL-6 levels were measured using ELISA kits (R&D Systems) according to the manufacturer’s instructions.

### Statistical Analysis

Data are expressed as mean ± SEM. Differences between groups were analyzed by Mann-Whitney U test, except cumulative incidence was evaluated with a χ^2^ test. A P value of less than 0.05 was considered statistically significant.
